# KBG syndrome complicated with chylothorax in a newborn: a case report and literature review

**DOI:** 10.3389/fped.2025.1690056

**Published:** 2025-10-28

**Authors:** Yuqian Wang, Xin Peng, Jing Zhu, Ning Zou, Xiaotong Yu, Liu Yang

**Affiliations:** ^1^ Department of Pediatrics, The Second Hospital of Dalian Medical University, Dalian, Liaoning, China; ^2^ Department of Neonatology, Dalian Women and Children’s Medical Group, Dalian, Liaoning, China

**Keywords:** KBG syndrome, chylothorax, *ANKRD11*, whole-exome sequencing, variant

## Abstract

**Objective:**

To discuss a unique case of KBG syndrome (KBGS) in neonates that developed congenital chylothorax and to examine how *ANKRD11* gene variations may be related to lymphatic malformation.

**Methods:**

The newborn was delivered at 38^+5^ weeks of gestation, presenting with congenital chylothorax, a ventricular septal defect, feeding difficulties, and craniofacial dysmorphism, and has been diagnosed with KBGS. Whole exome sequencing (WES) was employed to validate the diagnosis of a genetic disorder. Also, a systematic literature search of published KBGS cases between 1975 and June 2025 (*n* = 246) was carried out.

**Results:**

The newborn was found to have a heterozygous *ANKRD11* frameshift variant (NM_013275.6: c.37683769 del; p.His1256Glnfs *26), which came from his mother. The clinical presentation was congenital chylothorax, craniofacial dysmorphism (triangular face, bulging forehead, hypertelorism, short nose root, anteverted nostrils, big ears, and micrognathia), ventricular septal defect, and difficulty feeding. This was found to be the second case of KBGS with chylothorax in the literature review. The literature review revealed that the predominant universal neonatal phenotypes were feeding challenges (52.1%) and small-for-gestational-age status (41.1%). The long-term phenotypes included facial features (100%), macrodontia of upper central incisors (93.9%), skeletal disorders (90.7%), and developmental delay (93.2%). Other symptoms included short stature, neurological problems, and visual and auditory impairment. The incidence of skeletal developmental defects and developmental delay was considerably higher in truncated variant patients compared to missense variant patients (*p* < 0.05).

**Conclusion:**

The newborn presented with KBGS complicated by chylothorax, attributable to a pathogenic variant in the *ANKRD11* gene. These results broaden the existing knowledge of KBGS clinical and genetic spectra. WES or whole-genome sequencing should be important in diagnosing patients with unexplained developmental abnormalities. It is imperative that the management for KBGS is multidisciplinary to deliver optimal prognosis and long-term outcomes.

## 1 Introduction

KBGS (KBG syndrome) (OMIM #148050) is a rare multisystem dominantly inherited disorder first characterized by Herrmann in 1975, named after the initials of the three initial families documented (Keishi–Bukuryo–Gan). Its actual incidence is unknown, yet over 300 cases have happened throughout the world so far. The inheritance of the condition is autosomal dominant (1). Cases being reported are showing to be more in males than females, but the causal agent towards this sex distribution is yet not understood (2). KBGS is often underrepresented or misdiagnosed due to its broad phenotypic range and the overlap of certain symptoms with other neurodevelopmental disorders. Phelan-McDermid syndrome (PMS; OMIM #606232) is a rare condition characterized by unique facial features that bear resemblance to those observed in KBG syndrome (KBGS). Patients may exhibit characteristics such as ptosis (droopy eyelids), a broad nasal bridge (located between the eyes), and a bulbous nasal tip (3). PMS is caused by *SHANK3* gene haploinsufficiency as a result of deletions at the terminal region of chromosome 22q13.3 (4). In clinical practice, distinguishing between PMS and KBGS is crucial for accurate diagnosis. The breakthroughs in genetic testing have elucidated a molecular mechanism of it, and *ANKRD11* genetic defects that are pathogenic have been identified as the main cause of it using whole-exome sequencing (5). KBGS is most commonly caused by *de novo* pathogenic variants in the *ANKRD11* gene, although rare cases of familial inheritance have also been reported. In addition to single-gene variants, another known genetic mechanism is a microdeletion at chromosome 16q24.3 that includes *ANKRD11 (*6). Rarely, pathogenic variants in other genes—such as *KIAA1109*—have also been associated with KBGS. Notably, individuals with the 16q24.3 microdeletion may show distinctive brain abnormalities on neuroimaging, including hypoplasia of the corpus callosum (5) and cerebellar vermis (7). The presence of these findings during prenatal imaging could serve as an early diagnostic clue for KBGS.

KBGS has been clinically characterized by the craniofacial dysmorphism, especially in childhood. Some of its peculiarities are turned triangular face, enlarged forehead, hypertelorism, the short nasal root, anteverted nostrils, prominent ears, and mandibular hypoplasia (8). In students, the presence of macrodontia in permanent maxillary central incisors is a notable feature, observed in the majority of the KBGS cohort. This outlines the key dental characteristics of KBGS, which prompts the evaluation and has emerged as a significant element of clinical diagnosis. Feeding challenges can arise during the neonatal phase. Feeding difficulties in newborns may be due to congenital cardiac disorders such as ventricular septal defect or patent ductus arteriosus. Wide cranial sutures may also be observed. As children age, additional clinical manifestations may arise in certain cases, including neurodevelopmental disorders such as epilepsy, cognitive impairment, or global developmental delay; skeletal abnormalities; hearing loss; behavioral disturbances like attention deficit hyperactivity disorder and autism spectrum disorder; and distinctive dermatological and hair-related features (9). Additional abnormalities may encompass syndactyly (fused digits), enlarged earlobes, and associated morphological anomalies.

The clinical diagnostic criteria of KBGS was revised in 2016 to better recognize its heterogenous phenotype (10). The key features to be diagnosed are macrodontia of the maxillary central incisors, short stature, recurrent otitis media with or without hearing loss, and the presence of KBGS in a first degree relative. Secondary diagnostics include short fingers or other abnormalities of the hands, epilepsy, cryptorchidism, feeding problems, anomalies of the palate, autism spectrum disorder, and the late closure or enlargement of the anterior fontanelle. The presence of two major characteristics or one major characteristic and two minor characteristics in a patient with a development pathology, learning problems or behavioral aberrations allows making a clinical diagnosis. The necessity of molecular verification is evident, as the identification of *ANKRD11* variants can ultimately validate the clinical diagnosis. We present the case of a newborn boy who was diagnosed with KBGS with congenital chylothorax and present his clinical presentation and genetic report. The case also contributes to the KBGS studies because it brings out an unusual neonatal presentation and broadens the clinical and genetic spectrum of the disease.

## 2 Clinical report

The newborn had been presented with respiratory distress shortly after his birth. He was found to have moaning respirations as well as dyspnea, and therefore, he was admitted to the neonatal intensive care unit (NICU) of our hospital for further examination and the management.

**Figure 1:**
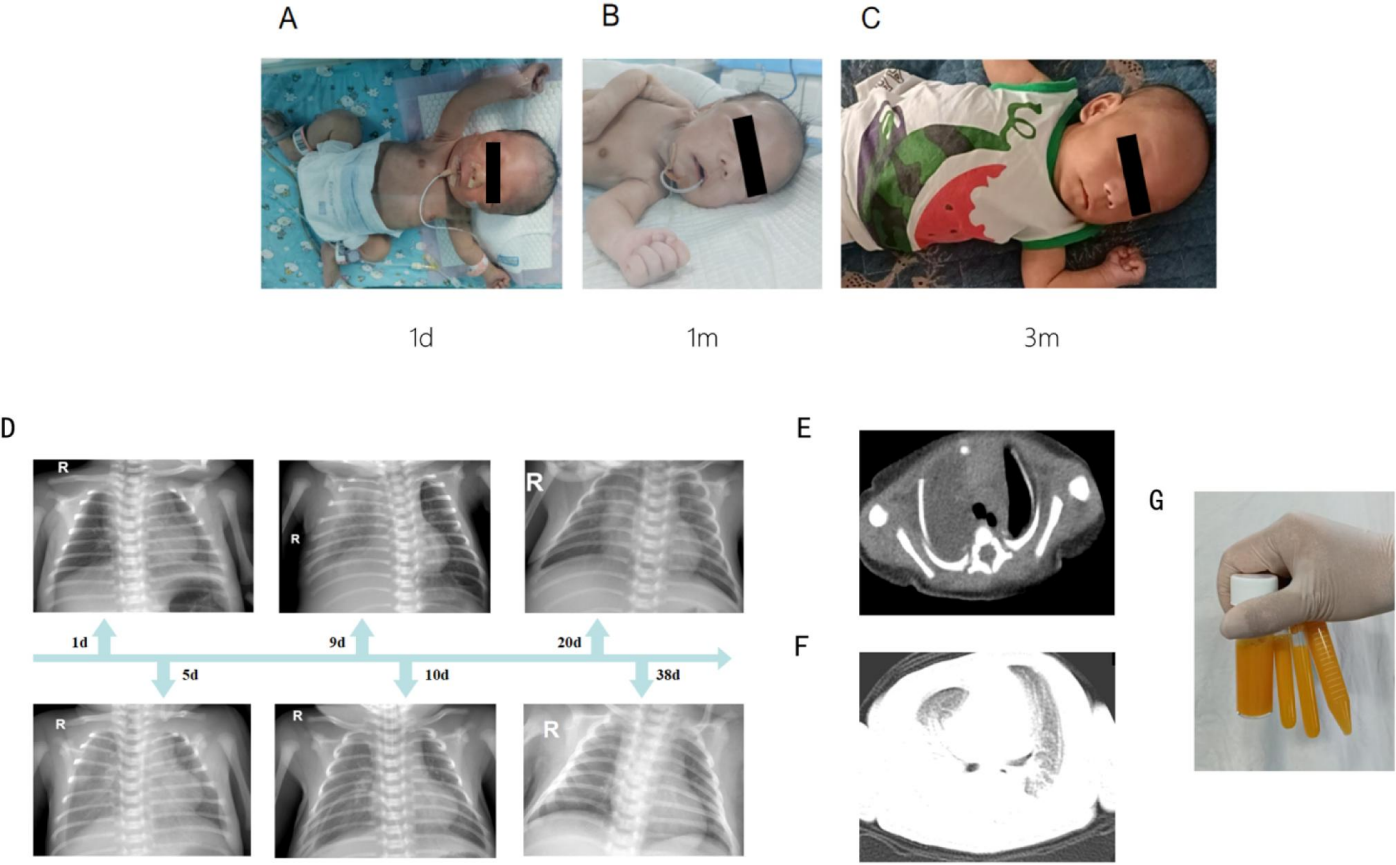
The characteristics of the newborn. **(A–C)** The characteristics of the newborn's craniofacial dysmorphism. **(D)** Continuous chest radiographs showed dynamic changes in the condition of the newborn. **(E,F)** Chest CT on day 9 of admissions demonstrated simple effusion with atelectasis of the right lung. **(G)** Pleural effusion is yellow chylous liquid.

**Figure 2:**
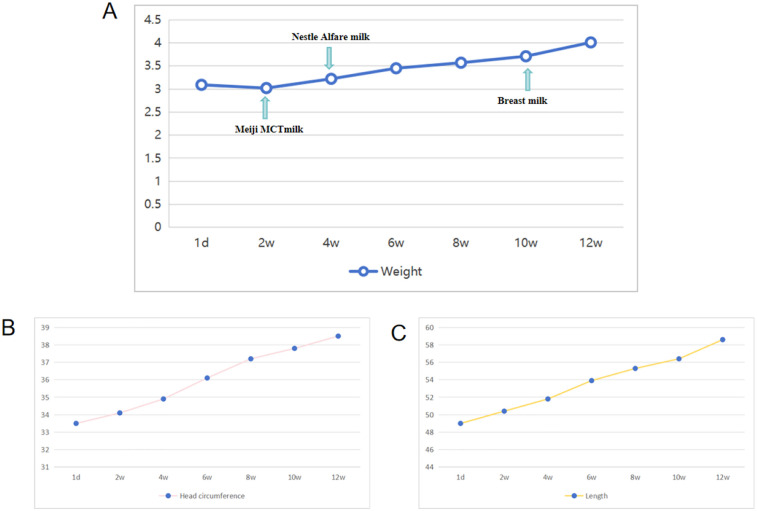
Weight, length, and head circumference were monitored within 3 months after birth. **(A)** Monitoring of weight in the patient. **(B)** Monitoring of head circumference in the patient. **(C)** Monitoring of length in the patient.

The newborn underwent a cesarean section at 38^+5^ weeks of gestation due to a scarred uterus. The mother was pregnant for the second time, resulting in the birth of her second child. The first baby is healthy and is a boy. The Apgar scores recorded at 1, 5, and 10 min were consistently 10. The brief perinatal course was uncomplicated, and the cord blood gas results indicated no abnormalities. The mother was healthy during pregnancy and did not have complications. No history was known of congenital anomalies or genetic disorders or other related syndromes. Significantly, the fetal ultrasound conducted at 34 weeks of gestation revealed indications of pleural effusion. Nonetheless, this anomaly was not identified in subsequent scans. The physical examination at the time of admission revealed dysmorphic features in the craniofacial region. These were straight eyebrows, hypertelorism, short nasal root, broad and prominent forehead, round nasal tip with anteverted nares, a full upper lip and micrognathia (Figures 1A–C). The testicles failed to descend into the scrotum. He exhibited hypotonia. The initial significant clinical problem of this newborn was dyspnea. Although non-invasive respiratory support was introduced immediately, the newborn improved but recurred in terms of the dyspnea. Serial chest radiographs demonstrated a gradual increase of pulmonary opacification of the right lung field (Figure 1D). The fetal color Doppler ultrasound indicated the presence of pleural effusion, which was confirmed by a subsequent chest color Doppler ultrasound on the second day post-birth, revealing 0.4 cm of pleural effusion. On the ninth day postnatally, his dyspnea progressively worsened. Chest CT on day 9 of admissions demonstrated simple effusion with atelectasis of the right lung (Figures 1E,F). Airway reconstruction experiments precluded airway malformations, whereas effusion depth was measured to 5.4 cm by means of pleural color Doppler ultrasonography. In the presence of progressive respiratory distress, endotracheal intubation and mechanical ventilation was performed together with continuous closed thoracic drainage. Analysis of the pleural fluid demonstrated 11,600 × 10^6 ^/L of nucleated cells where 90 percent were represented by lymphocytes. Sudan III (Li Fanta) stain was positive on pleural effusion and showed the presence of chylomicrons, chylothorax assay 3+ qualitative had positive result, which confirmed the presence of congenital chylothorax (Figure 1G). Somatostatin and erythromycin were added to the medical management in order to decrease chyle production. The volume of pleural effusion diminished progressively with the administration of these treatments. In the following weeks, the respiratory condition of the newborn was expected to show improvement. On the 12th day, a transition to non-invasive ventilation was implemented, leading to a successful weaning on the 17th day. Subsequent ultrasonography indicated a pleural effusion measuring less than 0.5 cm, observed 10 days prior to discharge, suggesting a positive response to treatment by the patient. Difficulty in feeding was the second important clinical issue. The baby could not perform effective oral sipping because of micrognathia and nor could he have properly latched on to the nipple. Moreover, poorly synchronized swallowing also decreased the efficiency of feeding. His sucking movement was significantly late compared with those of term neonates of the same gestational age. Consequently, the feeding of the nasogastric tubes was necessitated at birth. Intake could not be oral until the 30th day of life. At the beginning of the birth, the newborn consumed standard formula milk (Nestle). Enteral nutrition was adjusted after the development of chylothorax on day 9. The newborn was subsequently changed to a formula containing high-medium-chain triglyceride milk (MCT) (Meiji brand, MCT 98%, LCT 2%) and milk fortified with essential fatty acids (MCT 82%, LCT 18%). When clinically stable, he was transitioned to a partially hydrolyzed formula (Nestle Alesoft, MCT 40%, LCT 60%), and his long-chain triglycerides (LCT) were gradually reintroduced (Figure 2A). He later became able to endure exclusive breastfeeding. Monitoring of the growth indicated that the newborn weighed 3.04 kg (25–50th percentile) at birth. His weight was sluggish (Figure 2A). On three-month visit, his weight was 4.0 kg (<3rd percentile), whereas length and head circumference were within age-specific growth patterns (Figures 2B,C).

The newborn presents a significant issue, specifically congenital heart disease. An echocardiographic examination revealed congenital heart disease characterized by a left-right shunt, specifically a 5.0 mm ventricular septal defect (VSD) and a 3.0 mm patent foramen ovale (PFO). The newborn underwent additional examinations during the hospital stay. The automatic auditory brainstem response (AABR) indicated a failure in binaural hearing, raising concerns regarding the potential implications of his congenital hearing impairment. Cranial magnetic resonance imaging (MRI), abdominal ultrasonography (liver, gallbladder, and spleen), and a fundus examination showed no abnormalities. Serially measured blood gases and lactic acid were in the normal limits during the course of admission. Blood ammonia level: 24 umol/L. Blood glucose levels were within the normal range. One month post-birth, the Test of Infant Motor Performance (TIMP) assessment indicated a percentile ranking of 10%–16%.

**Figure 3:**
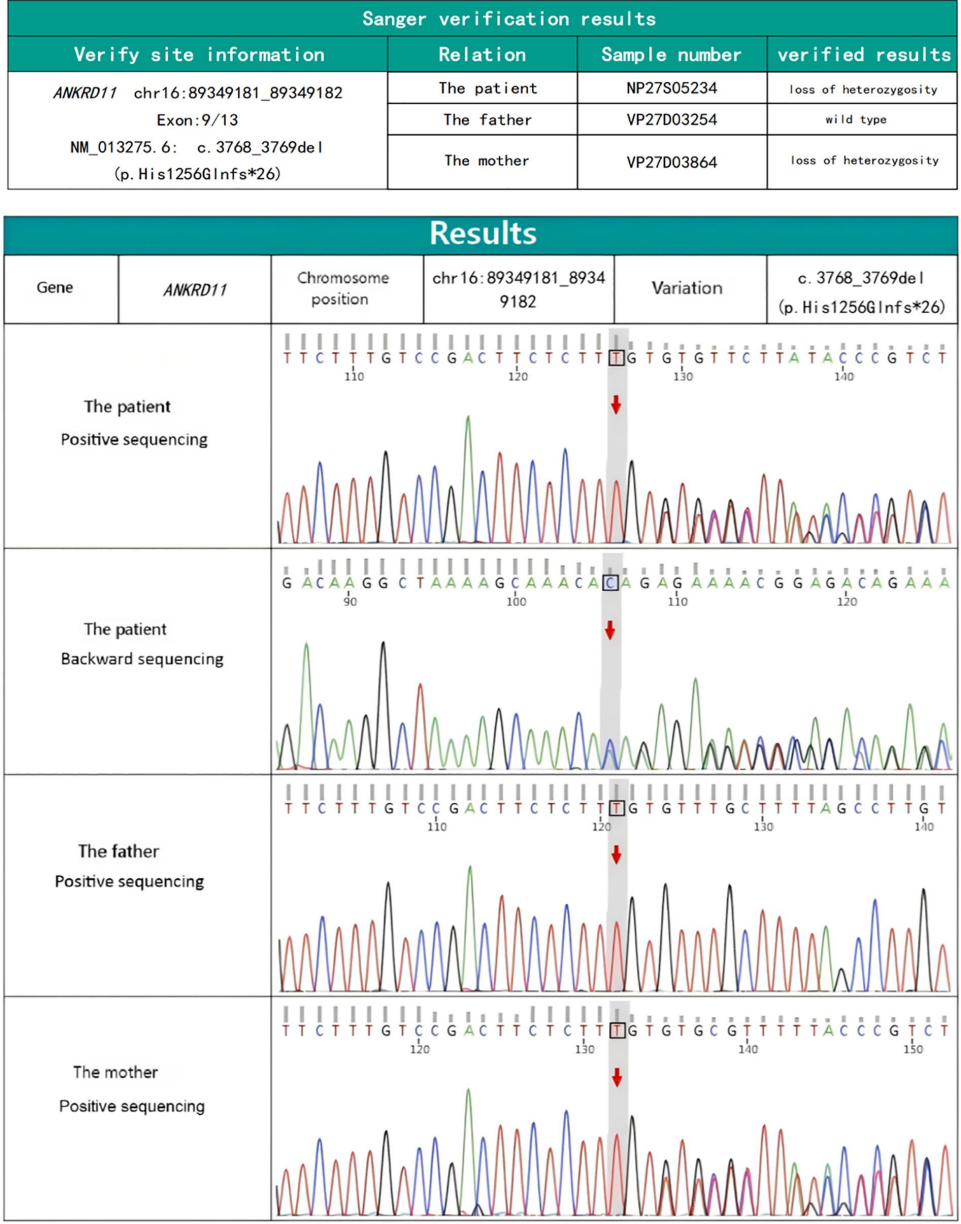
*ANKRD11* gene sequencing map of the newborn and his parents.

Characteristic craniofacial features together with feeding difficulties, congenital chylothorax and congenital heart disease suggested the possibility of underlying genetically-based syndrome. To better explore the pathogenesis, 2 mL of peripheral venous blood was drawn to analyze cytogenetic and conduct a whole-exome sequencing (WES) (tested by KingMed Diagnostics, China). The chromosomal structure and number were normal as analyzed by karyotype. WES, however, detected a potentially deleterious pathogenic, heterozygous frameshift variant in *ANKRD11* gene (NM_013275.6: c.3768_3769del; p.His1256Glnfs*26) (Figure 3). The pathogenic variant was inherited from the mother. The newborn was diagnosed with KBG syndrome based on the revised diagnostic criteria of KBGS established in 2016, in conjunction with the pathogenic variant of the *ANKRD11* gene.

At the time of reporting, the patient is three months old and is under close follow up. The constant evaluations are aimed at growth and diet, hearing, and nervous system development. Multidisciplinary interventions that provide a combination of cardiology, nutrition, audiology, and genetic counseling have also been preemptively put in place in a bid to minimize long term adverse outcomes.

### 2.1 Literature retrieval strategy

A detailed literature search was done to systematically review the clinical characteristics and the genetic case of KBG syndrome (KBGS). Keywords were: KBG, *ANKRD11* and 16q24.3 microdeletion/microduplication. These were used in several electronic databases, including PubMed, Ovid Medline, SpringerLink and Chinese Biomedical Literature Database (CBMD). The search has been carried out between the origination of the first description of KBGS in the past to the month of June in the year 2025. All primary studies documenting human cases were incorporated; however, guidelines and expert consensus, narrative or systematic reviews, and studies involving animals were excluded. Strict inclusion criteria were used to guarantee the reliability. The cases had to fulfill the following requirements: (i) a reasonably comprehensive clinical data; and (ii) diagnosis that was based on molecular genetic data, viz., the presence of either pathogenic variants in the *ANKRD11* gene or 16q24.3 microdeletions. The following studies were excluded: studies based on incomplete phenotype data or unconfirmed diagnoses; studies that lacked genetic information. In each instance, data was collected concerning clinical presentation, genetic outcomes, inheritance patterns, and long-term follow-up when available. The obtainable data would be synthesized in order to produce complete overview of both neonatal and non-neonatal phenotypes related to KBGS. The primary objective was to identify atypical or rare traits that could enhance the understanding of the syndrome.

### 2.2 Statistical processing

After data extraction, a pooled analysis was done by combining all the eligible cases in the literature with the current case. Patients were also classified into two categories in accordance with their genetic results: truncated variation group (containing nonsense and frameshifting variants that caused early termination of the protein expression) and missense variation group (refers to single amino acid substitutions with no premature termination of protein sequence). This sectioning was needed based on the fact that prior reports indicated that there might be genotype-phenotype correlations in KBGS though the proof are still scarce.

The primary variables of focus included key neonatal characteristics such as feeding difficulties, small-for-gestational-age status, congenital heart disease, palatal defects, and cryptorchidism, alongside the long-term consequences encompassing craniofacial dysmorphism, dental abnormalities, skeletal abnormalities, developmental delay, neurological manifestations, and visual and auditory deficiencies.

The chi-square (*x*^2^) test was used to make comparisons of clinical characteristics among the groups. Fisher exact test was used when >20% of the cells of a contingency table had a predicted frequency of <5. The statistical significance of the *p*-value was set at <0.05 and may be interpreted as the possibility of differences in the two genetic groups. The current case was classified into truncated variation group that was analyzed, since sequencing showed a frameshift deletion in *ANKRD11*.

### 2.3 Literature retrieval results and analysis

**Figure 4:**
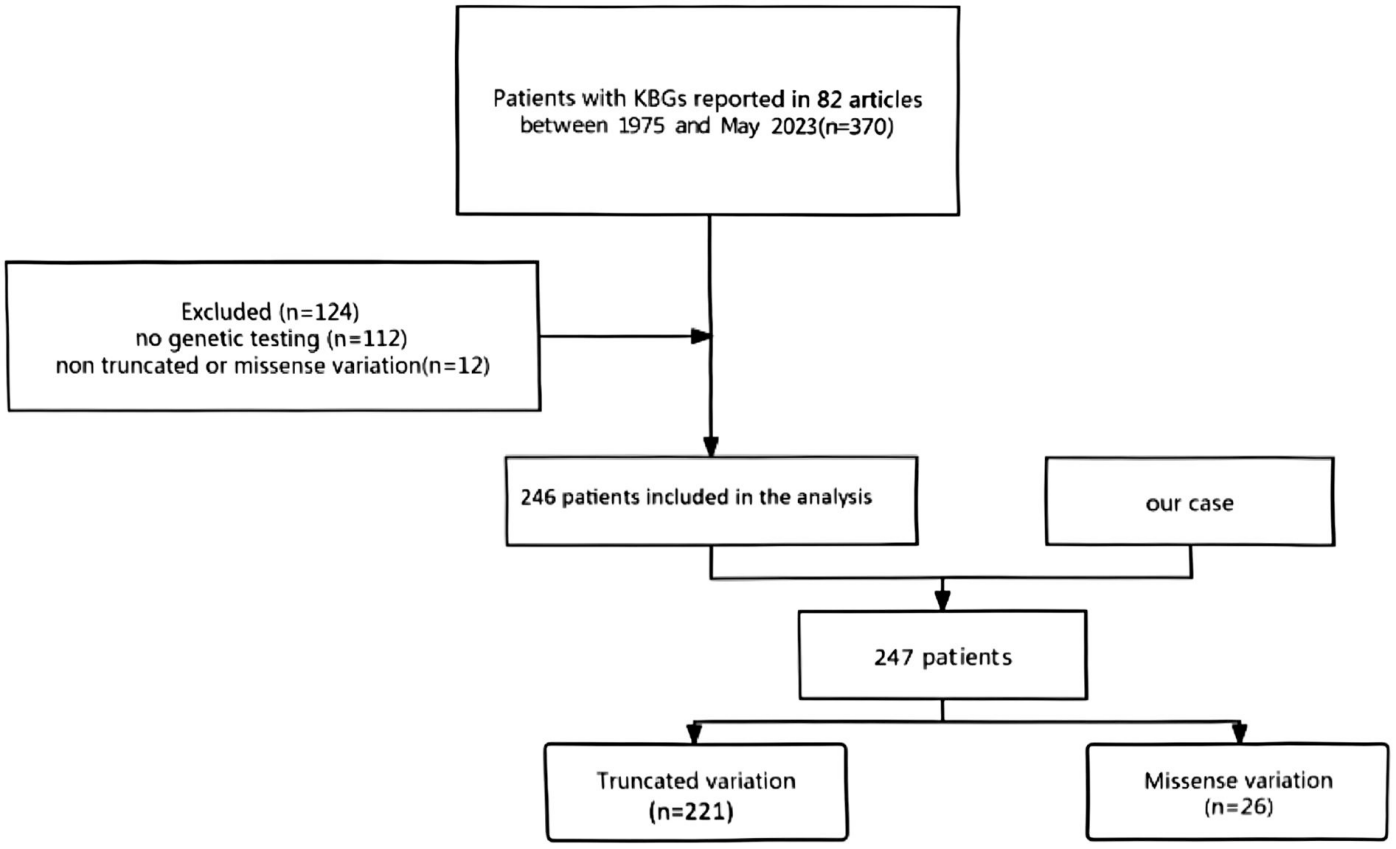
The process of screening the patients.

A total of 370 patients were reported in the 82 identified relevant publications. Upon eliminating 124 articles with incomplete genetic information, with only reviews listed in them, or with patients without established truncating or missense changes, 246 case studies fit the strict inclusion criterion (Figure 4). Including the previous patient, this provided a study population of 247 individuals diagnosed with KBGS and confirmed to express pathogenic *ANKRD11* variants. Of these, 221 patients had truncating variants (frameshift and nonsense) whereas 26 patients had missense variants.

Phenotypic data analysis found neonatal manifestations to vary though follow a number of common trends. Feeding difficulties were most frequently encountered among neonates (52.1%) and were the greatest number of early symptoms. Small-for-gestational-age status (41.1%), cryptorchidism (39.0%), and palatal defects (37.3%) are other commonly noted histopathologic features in a neonate. Also in 29.1 percent cases, congenital heart disease was diagnosed, most frequently ventricular septal defect or patent ductus arteriosus. These results underscore the clinical heterogeneity of KBGS during the neonatal stage and the possibility of under-recognition in cases in which craniofacial or dental features have not yet manifested.

In contrast, there was much more uniformity in non-neonatal phenotypes. Nearly all (100%) patients showed a typical craniofacial appearance that comes up as a hallmark of KBGS. The stomatognathic anomaly was stated as dental abnormalities with macrodontia in the maxillary central incisors in 93.9 percent of the cases. Ninety percent of the patients exhibited skeletal developmental anomalies e.g., delayed bone age, joint malformations, or deformity of the thorax. Developmental delays–in cognitive, language, motor and behavioral delays were seen in 93.2%. Other findings noted in over a third of the patients were short stature, neurologic manifestations (epilepsy, abnormal electroencephalography, or neuroimaging results), eye abnormalities, and hearing loss. This information supports the conclusion that the phenotype can differ at the neonatal stage, but long-term outcomes show a high phenotypic content consistency (Table 1).

**Table 1:** Analysis of clinical phenotype frequency of ANKRD11 variants.

Period	Clinical features	Analysis of clinical phenotype frequency of ANKRD11 variants (*n* = 247)			
		*n*	Positive	Negative	Incidence rate
Neonatal period	SGA	17	7	10	0.411
	Feeding difficulties	23	12	11	0.521
	Palatal irregularity	150	56	94	0.373
	Congenital heart disease	182	53	129	0.291
	Cryptorchidism	82	32	50	0.390
Non-neonatal period	Craniofacial anomalies	178	178	0	1.000
	Dental anomalies	179	168	11	0.938
	Skeletal anomalies	216	196	20	0.907
	Short stature	221	111	110	0.502
	Growth retardation	220	205	15	0.931
	Abnormality of the nervous system	139	48	91	0.345
	Ocular anomalies	147	69	78	0.469
	Hearing loss	188	66	122	0.351

Statistical testing of the two genetic subgroups revealed there was no significant difference between patients with truncated with vs. missense variants in the neonatal presentation. But during the non-neonatal stage there were distinct differentials. Specifically, there was a high prevalence of skeletal developmental abnormalities and developmental delay in the truncated variant patients significantly more prevalent than in the missense variant patients (*p* < 0.05). The other phenotypes such as craniofacial dysmorphism, dental abnormalities as well as neurological features were represented with the same rate in the two groups. These results indicate that truncating and missense variants can cause both skeletal and neurodevelopmental morbidity, yet potentially in different degrees (Table 2).

**Table 2:** Frequency of clinical phenotypes between truncated variation and missense variation of *ANKRD11.*

Period	Clinical features	Truncated variation (*n* = 221)			Missense variation (*n* = 26)			
		*n*	Positive	Negative	*n*	Positive	Negative	*P*
Neonatal period	SGA	13	5	8	4	2	2	1.000
	Feeding difficulties	12	5	7	11	7	4	0.413
	Palatal irregularity	132	46	86	18	10	8	1.000
	Congenital heart disease	163	45	118	19	8	11	0.193
	Cryptorchidism	63	28	35	19	4	15	0.106
Non-neonatal period	Craniofacial anomalies	153	153	0	25	25	0	1.000
	Dental anomalies	159	150	9	20	18	2	0.354
	Skeletal anomalies	198	186	12	18	10	8	<0.01
	Short stature	195	96	99	26	15	11	0.532
	Growth retardation	195	185	10	25	20	5	0.017
	Abnormality of the nervous system	122	45	77	17	3	14	0.173
	Ocular anomalies	127	59	68	20	10	10	0.813
	Hearing loss	167	60	107	21	6	15	0.632

To conclude, the literature review and the statistical comparison confirm that the conclusion is, clinically, KBGS is heterogeneous disorder with consistent long-term phenotypes but heterogeneous presentations as neonates. Recent discoveries of genotype phenotype correlations are of great value in diagnosis, prognosis, and genetic counseling.

## 3 Discussion

KBG syndrome (KBGS) is a rare autosomal dominant hereditary disease that affects several organ systems. Specific craniofacial features are often presented, which makes recognition in the clinic possible, especially to the nervous and skeleton systems. *ANKRD11* chromosomal deletion and loss sequence variants of the functional sequence have been associated with the pathogenesis of KBGS, yet the molecular pathogenesis behind it remains to be elucidated. The *ANKRD11* gene encodes the protein with several ankyrin repeat domains (11), five ankyrin domains, two inhibitory domains, and one activation domain (12). It is broadly expressed throughout human tissues, and is particularly expressed in the brain, where it is localized within the neuronal and glial nuclei (13). This pattern of expression reveals its strong significance in the development of the nervous system, especially in terms of neuronal migration and differentiation.

Molecular analysis has revealed *ANKRD11* to control gene expression through association with nuclear receptor coactivators and nuclear histone deacetylase corepressors in regulation of transcriptional activation. Moreover, it regulates histone acetylation in neuronal development, which is one of the mechanisms that may elucidate cognitive malfunction in children with KBGS caused by pathogenic variants (14). *ANKRD11* variants are also associated with KBGS as well as with various other neurodevelopmental disorders such as autism spectrum disorder and intellectual disability. *in vitro* experiments demonstrate that *ANKRD11*-deleted neurons have impaired dendrite development, decreased complexity as well as dendritic spine shape change (15). One way such defects can be used to explain the prevalence of developmental delay, learning difficulties, and abnormal behavior among the affected children, is to offer a biological explanation.

Efficiency of *ANKRD11* is not restricted to the nervous system. Studies indicate that its interference also extends to the chondrocyte differentiation in the growth plate impairing longitudinal growth in the bones (16). The patients, therefore, manifest short stature and skeletal dysplasia (17). In fact, comparison of populations clearly shows that children with KBGS are of heights that are often two standard deviations below that of age-matched normal (18). *ANKRD11* also plays a role as a transcriptional co-activator of p53 and seems to increase its acetylation and transcriptional activity (19). Due to the involvement of p53 signaling in the craniofacial development, disrupted *ANKRD11*-p53 interactions are a probable cause of characteristic craniofacial morphology of KBGS patients (20).

These molecular mechanisms create concerns regarding the risk of cancer, as well. Isrie et al. postulated that KBGS patients might be predisposed to tumorigenesis (21). In line with this hypothesis, cases have been reported of patients with 16q24.3 microdeletion developing unusual-types of cancers including paratesticular rhabdomyoma (22). The other case reported a 12-year-old female patient with KBGS that presented acute myeloid leukemia (23). Even though the findings still suggest an anecdotal finding, it underscores the importance of serial monitoring of KBGS patients, especially the subset that harbor specific *ANKRD11* variants.

Our current case had a heterozygous deleted frameshift transition in *ANKRD11* (NM_013275.6: c.3768_3769del; p.His1256Glnfs*26). The variant is anticipated to terminate the C-terminal functional domain which communicates with nuclear receptor regulators, hence, hindering the transcriptional control. This mutation originated from the maternal lineage. Nevertheless, the mother of the affected child did not perform comprehensive exome gene sequencing to ascertain if she was a KBGS patient. The incomplete penetrance that characterizes KBGS is an additional factor that warrants consideration. The phenotype of our case was not fully defined. It has been observed that incomplete penetrance occurs in approximately 18 percent of families affected by KBGS. Historically, it was thought that KBGS was predominantly caused by *de novo* variants; however, accumulating evidence suggests that familial inheritance with incomplete penetrance may be more common than previously acknowledged.

The correlations between genotypes and phenotypes are also clinically significant. We found that truncating variants, including nondescript or frameshift respectively, are linked with harsher neurodevelopmental effects. These substantiate the usefulness of Molecular subtype classification during genetic counseling and prognosis. The clinical implication of the truncation variants by clinicians in terms of anticipatory guidance should therefore be noted. The variant type in our case is a truncated variant. Consequently, it is essential for physicians to monitor his neurodevelopment throughout the follow-up process.

Most notable clinical neonatal presentation of KBGS is difficulty in feeding. This can be due to craniofacial deformities like micrognathia, poor coordination of sucking and swallowing and diffuse hypotonia. Characteristic craniofacial appearance (triangular or round in shape, straight eyebrows, eyes wide-set, short root of the nose, anteverted nares, and full upper lip) is frequently identified during the prenatal or retronatal period and can also be used early to establish a diagnosis. Approximately one-third of patients are reported to have congenital heart disease, especially a mix of the most common congenital heart diseases, namely ventricular septal defect and patent ductus arteriosus. Another common manifestation that may not be immediately noticed is hearing loss that is usually detected in the hearing of newborns during hearing screening (24).

Our literature review identifies three principal features in these neonatal cases. First, 52.1% experienced complications related to feeding, with several individuals requiring nasogastric feeding interventions. Second, the determination of Small-for-gestational-age status revealed a prevalence of 41.1%. Third, cryptorchidism was observed in 39% of the patients. Collectively, these findings underscore the importance of evaluating for an underlying genetic syndrome, particularly KBGS, in neonates presenting with feeding difficulties, Small-for-gestational-age status, and/or distinctive facial phenotypes. Respiratory distress was a distinguishing and rare aspect of the disease; prior, a case report revealed a case of neonatal KBGS associated with chylothorax (25). Facial dysmorphism was observed in all non-Neonatal cases, establishing facial deformity as the primary criterion for diagnosing KBGS. Differential diagnosis is essential since the phenotypic range of KBGS is shared with other disease variants. As an example, Sotos syndrome (OMIM #117550) is characterized by intellectual disability and characteristic craniofacial appearance (26), whereas Noonan syndrome (OMIM #601321) can be associated with congenital heart disease, short stature and facial dysmorphism (27). It might be difficult to distinguish between these conditions based on clinical grounds only and genomic testing is important. A conclusive diagnosis can be delivered through whole-exome sequencing or whole-genome sequencing that assists in proper management and counseling of the family. Early genetic testing is specifically auspicious in predicting complications, designing follow-up approaches, and enhancing long-term results.

Overall, our results emphasize that KBG syndrome (KBGS) is a multifactorial and presumably heterogeneous condition with wide phenotypic range, mostly including well-known symptoms but with new emerging features like congenital chylothorax. Notably, the case of chylothorax that we observed in our neonate broadens the clinical spectrum of KBGS and offers novel possibilities of the role of the lymphatic system in its mechanism of action. Although limited data relating to lymphatic complications in KBGS have been reported, our case points to the fact that an early loss of *ANKRD11* functional properties is likely to impair lymphatic development and cause uncommon yet clinically significant complications. A more systematic approach to reporting these cases will be essential in determining if congenital chylothorax represents a recurrent, yet under-recognized, phenotype of KBGS. In addition, the variable disease presentation supports the importance of extensive genetic testing, particularly whole exome sequencing (WES) in neonates with atypical syndromic associations of dysmorphic features as well as feeding or respiratory or lymphatic complications of unclear cause. Use of clinical features alone may result in either misdiagnosis or delay in the diagnosis since there is a significant overlap between KBGS and other conditions (e.g., Sotos or Noonan syndrome). WES facilitates the accurate diagnosis and empowers patients and their families to engage in early discussions regarding prognosis, allowing them to formulate further plans and adopt appropriate perspectives.

KBGS treatment is largely supportive in nature by dealing with the multisystem complications which occur in the course of life (28–30). Cross functional teamwork is also important where geneticists, neonatologists, orthopedists, neurologists, endocrinologists, cardiologists, and speech or occupational therapists would be involved depending on the needs of the patient. An example is that craniofacial anomalies could be evaluated surgically whereas consistent feeding problems could require nutritional input or gastrostomy support. Behavioral and cognitive issues can be tackled with the help of special education, early intervention programs and psychiatric or behavioral treatment in case it is necessary. From a longitudinal viewpoint, surveillance is of paramount significance, as children with KBGS generally demonstrate minimal spontaneous catch-up growth in their adult years (18). Furthermore, mutations in the *ANKRD11* gene could be linked to the progression of tumors in certain instances. Consequently, it is crucial to ensure thorough monitoring during the implementation of intervention measures, such as the administration of growth hormones (28).

In fact, the case with our patient, whose pathogenic variant came from his mother. It underlines the intricacy of genetic counseling in involved families by showing that unaffected carriers might be present in spite of carrying truncating or deleterious variants. It only supports greater caution in interpreting family data and provides further support that family studies have to outline the likelihood of phenotypic variability in predictive testing to at risk family members. This complication also highlights the predictive value of molecular subtype analysis, in which truncating variants are linked to a more impairing neurodevelopmentic outcome than missense variants. These differences can be useful to stratify the risk and inform early interventions.

To the future, it is expected that studies on the molecular mechanisms of *ANKRD11* and its contribution to chromatin conformations, neuronal migration, and skeletal growth will expand, and future therapeutic opportunities will be discovered. Mechanistic insights into the functional loss of *ANKRD11* may be elucidated by functional animal model studies how *ANKRD11* dysfunction derails lymphatic development and leads to complications such as chylothorax. The availability of genomic sequencing is increasing, and greater cohorts of KBGS patients can be recruited to better characterize genotype-phenotype correlations, refine diagnosis and further tailor management strategies. Ultimately, these advances hold promise for developing more personalized approaches to care, improving long-term outcomes and quality of life for individuals affected by KBGS.

## 4 Conclusion

This article reports a case of a full-term neonate diagnosed with KBG syndrome during the neonatal period, complicated by congenital chylothorax. The detection of a heterozygous frameshift variant in the *ANKRD11* gene further underscores its highly incomplete penetrance and provides a clear molecular basis for clinical symptom assessment. Although congenital chylothorax is rare, it may serve as an important early warning sign of KBGS in neonates. In addition to feeding difficulties and distinctive craniofacial features, such manifestations may contribute to the early recognition of this condition.

For newborns presenting with unexplained pleural effusion in combination with dysmorphic features and feeding challenges, WES or whole-genome sequencing should be strongly considered to achieve timely diagnosis and intervention. As complications are a major determinant of long-term quality of life in KBGS, early identification plays a critical role in optimizing care. At present, there is no targeted therapy for KBGS, and management remains largely symptomatic. Therefore, multidisciplinary collaboration (including encompassing neonatology, genetics, orthopedics, endocrinology, neurology, and psychology) is essential for tailoring individualized management strategies, minimizing complications, and supporting growth and neurodevelopment.

Furthermore, long-term follow-up is indispensable to monitor disease progression and provide anticipatory guidance for families. Prenatal diagnostics and genetic counseling are also very important. Early clarification of possible neurodevelopmental consequences, as well as an understanding of the inheritance pattern, enables informed decision-making and risk assessment for affected families. Collectively, these measures contribute to improving clinical outcomes and quality of life for patients with KBGS.

## Data Availability

The raw data supporting the conclusions of this article will be made available by the authors, without undue reservation.
